# Interleukin-34-regulated T-cell responses in rheumatoid arthritis

**DOI:** 10.3389/fmed.2022.1078350

**Published:** 2022-11-30

**Authors:** Hye Eun Park, Hanna Oh, Jea-Hyun Baek

**Affiliations:** School of Life Science, Handong Global University, Pohang, South Korea

**Keywords:** rheumatoid arthritis, macrophage, T-cell, IL-34, inflammation

## Abstract

Rheumatoid arthritis (RA) is a chronic autoimmune disease with a multifaceted etiology, which primarily affects and results in the deterioration of the synovium of patients. While the exact etiology of RA is still largely unknown, there is growing interest in the cytokine interleukin-34 (IL-34) as a driver or modulator of RA pathogenesis on the grounds that IL-34 is drastically increased in the serum and synovium of RA patients. Several studies have so far revealed the relationship between IL-34 levels and RA disease progression. Nevertheless, the significance and role of IL-34 in RA have remained ambiguous, as illustrated by two most recent studies, which reported contrasting effects of genetic IL-34 deletion in RA. Of note, IL-34 is a macrophage growth factor and is increasingly perceived as a master regulator of T-cell responses in RA *via* macrophage-dependent as well as T cell-intrinsic mechanisms. In this regard, several studies have demonstrated that IL-34 potentiates helper T-cell (Th) responses in RA, whereas studies also suggested that IL-34 alleviates synovial inflammation, potentially by inducing regulatory T-cells (Treg). Herein, we provide an overview of the current understanding of IL-34 involvement in RA and outline IL-34-mediated mechanisms in regulating T-cell responses in RA.

## Introduction

Rheumatoid arthritis (RA) is an autoimmune disease characterized by synovial inflammation attributed to an abundance of inflammatory cytokines and leukocytes, leading to the destruction of surrounding bone and cartilage (1–4). Symptoms of RA include joint pain, swelling and/or stiffness, and rheumatoid nodules under the skin (5). RA synovial inflammation involves self-reactive immune responses triggered by both innate and adaptive immune cells (e.g., neutrophils, macrophages, and T lymphocytes) (6–8). Notably, the inflamed synovium is continuously infiltrated by neutrophils, which are cleared by synovial macrophages (7–9). In general, activated macrophages may have versatile roles being ‘healers' and ‘destroyers' and are commonly divided into (1) classically (M1) and (2) alternatively activated macrophages (M2) being pro-inflammatory and anti-inflammatory cells, respectively (9). In the RA synovium, macrophages have been demonstrated to induce the proliferation of fibroblast-like synoviocytes (FLS), recruit monocytes, and activate T helper (Th) cells (e.g., Th17) (10, 11), collectively perpetuating the cycle of joint destruction in RA (12, 13). In contrast, other studies showed that macrophages counteract inflammation and clinical symptoms in RA (9). Overall, the pathological roles of macrophages in RA are enigmatic and need to be further clarified.

Interleukin-34 (IL-34) is a macrophage growth factor, which is detected at high levels in the synovial fluid (SF), synovial cells, and serum of RA patients (14, 15). Synovial IL-34 levels are implicated in leukocyte accumulation (16). IL-34 is also proposed as a biomarker of RA remission due to a strong correlation between IL-34 and the relapse of RA after the termination of the anti-rheumatic drug treatment (4, 17–23). While IL-34 presence in different states of RA pathology has been described, the immunological role of IL-34 has not been yet clearly delineated. Although systemic IL-34 was shown to exacerbate RA (11, 24), the deleterious role of IL-34 in RA has remained controversial through various studies including the latest finding suggesting that IL-34 is beneficial in the effector phase of RA (9). Whether IL-34 takes on either a pro- or anti-inflammatory role, is ambiguous and may depend on pathologic conditions as studies of certain diseases (e.g., acute kidney injury, inflammatory bowel disease, lupus nephritis, Sjögren's syndrome) characterized IL-34 as a pro-inflammatory factor (25–29), while other clinical settings (e.g., atopic dermatitis, autoimmune hepatitis, lung cancer, sepsis) identified IL-34 as a mediator of anti-inflammatory responses (30–33). Recently, accumulating evidence has suggested that IL-34-dependent pathologic mechanisms in RA involve T cells, and IL-34 substantially controls the nature of T-cell responses in RA. However, the current conceptualization of IL-34-mediated T-cell regulation in RA is as vague as the proposed immunological roles of IL-34 (9, 11, 34). Therefore, there is a strong need to amalgamate information surrounding IL-34 involvement in RA pathogenesis, especially regarding how IL-34 integrates into the mechanisms of T-cell activation noted in RA.

## IL-34 in RA

**Figure 1:**
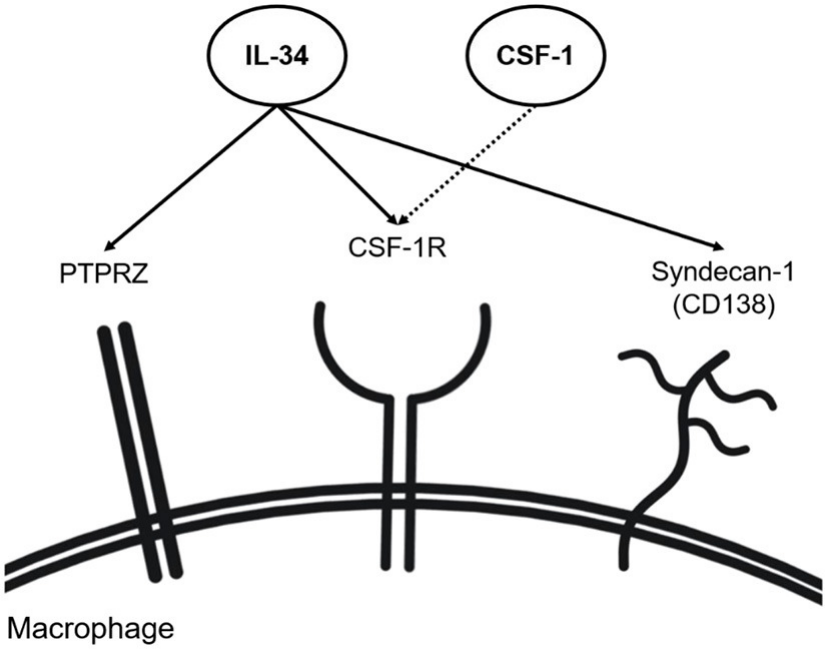
IL-34 receptors (including co-receptors). IL-34 has shown the capability of binding to CSF-1R alongside CSF-1 on macrophages. Independent of CSF-1, IL-34 has shown binding to syndecan-1 and PTPRZ found on the surface of macrophages.

IL-34 is a cytokine, which binds to multiple receptors. The colony-stimulating factor-1 receptor (CSF-1R) is the first identified and best-known receptor for IL-34. CSF-1R is expressed on the surfaces of mononuclear phagocytes (e.g., macrophages) and is activated by two known ligands: CSF-1 and IL-34 (Figure 1) (35). CSF-1R is crucial for the differentiation, polarization, activation, and maintenance of mononuclear phagocytes (2, 36–41). IL-34 and CSF-1 may have non-redundant roles in RA as studies have reported unique pathological properties of IL-34 that are independent of CSF-1 (25, 37). Interestingly, IL-34 is more drastically upregulated in human and murine RA as compared to CSF-1, underlining a predominant role of IL-34 in CSF-1R signaling during the pathogenesis of RA (9). Studies have investigated the effects of CSF-1R inhibition in RA. A study, using both human and murine RA models, reported suppression of RA inflammation when CSF-1 and IL-34 were simultaneously inhibited, while individual neutralization of either factor did not demonstrate beneficial effects in RA models (36). Nevertheless, numerous studies pointed to the direct association between IL-34 and RA pathology, although the effects of IL-34 on RA were not always consistent (2, 4, 9, 11, 19, 24, 41–44). For example, a very recent study demonstrated that the genetic deletion of IL-34 aggravates the effector phase of RA, implicating a protective role of IL-34 in RA (9), while another study showed that the genetic deletion of IL-34 mitigates RA pathology (11).

IL-34 has been recently associated with metabolism in RA-associated macrophages. IL-34 may metabolically reprogram macrophages toward a hyper-glycolytic M1-like phenotype upregulating glucose transporter 1 (GLUT1), mammalian target of rapamycin (mTOR), and hypoxia-induced factor 1α (HIF-1α) in the RA synovium (11, 43); however, we need further clarification on whether this is a general mechanism of IL-34, applicable to any physiological or pathological condition.

CSF-1R is not only linked to macrophage activity in RA but is also expressed by RA FLS (4). Increased IL-6 production by the RA FLS was noted during stimulation with IL-34 in a CSF-1R-dependent manner (4). Also, synovial fibroblasts may conceivably function in the RA synovium through IL-34-mediated pathways as evidenced by a study showing that IL-34 supports synovial fibroblast survival by the activation of the STAT3-miR-21 axis (17).

Not only do FLS or synovial fibroblasts respond to exogenous IL-34 expressing relevant T-cell surface receptors, but it is also likely that they are the major source of IL-34 in RA SF. Indeed, in the presence of TNF-α, FLS upregulate the expression of IL-34 (20). Similarly, a different study showed that synovial fibroblasts increase the expression of IL-34 after exposure to TNF-α and IL-1β (45). Of note, synovial macrophages likely produce and provide TNF-α and IL-1β to synovial fibroblasts (46, 47). Synovial fibroblasts may, in turn, express IL-34 and ultimately stimulate Th17 cells to express IL-17 (48).

In addition to the activation of macrophages, T cells, and FLS, IL-34 may promote synovial inflammation by the initiation of osteoclastogenesis (43). Of note, osteoclastogenesis and osteoporosis are prominent in synovial inflammation (49–52). Indeed, the imbalance between M1 and M2 macrophages in RA patients has been linked to increased occurrence of osteoclastogenesis in RA patients (16). Importantly, IL-34 is known to directly promote osteoclast formation like CSF-1 (53) and may upregulate the expression of the receptor activator of nuclear factor (NF)-κB-ligand (RANKL) for the progression of osteoclastogenesis in RA (21, 53–55).

In addition to CSF-1R, syndecan-1 (CD138) and protein tyrosine phosphatase receptor type ζ1 (PTPRZ1) bind IL-34 (Figure 1). Syndecan-1 is a cell surface proteoglycan functioning as a co-receptor to various cell surface receptors (56) and, besides, a regulatory factor involved in cell adhesion and cell migration (49–52). Syndecan-1 is present in various human subsets of monocytes and macrophages (57). On macrophages, syndecan-1 enhances the activation of IL-34 receptors and increases their motility (58). In line with this, another study showed that the migration of syndecan-1-expressing monocytes and macrophages is induced with IL-34 and reversely inhibited by anti-syndecan-1 neutralizing antibodies (56). Syndecan-1 has been also associated with various diseases, such as aortic aneurysms (52), cancer (59), dermatitis (60), and multiple myeloma (61). Expression of both IL-34 and syndecan-1 was reported to be elevated in RA synovium (11, 43). As such, the syndecan-1 expression for the binding of IL-34 has recently emerged as a molecule of interest in RA. Although the exact mechanism of the interaction between IL-34 and syndecan-1 remains elusive, it was shown that the lack of syndecan-1 impairs macrophage differentiation, ultimately attenuating murine IL-34-induced arthritis (11, 43). Syndecan-1 was detected in the mononuclear infiltrates of the sub-lining layer in the synovium of RA patients, thus posing the possible role of syndecan-1 in RA pathophysiology in the migration and maintenance of mononuclear phagocytes in the synovium (62). A study detecting syndecan-1 mRNA expression within the joints in a murine RA model showed that syndecan-1 mRNA expression levels are highest in aging knee joints, implying its role in age-related RA (63). Besides impacting macrophage functions and synovial inflammation, syndecan-1 induces osteoclastogenesis, resulting in the degradation of joint and bone structures in RA patients. Syndecan-1 levels are positively correlated with levels of RANKL in murine serum while being negatively correlated with serum osteoprotegerin (OPG) (64). The study linked increased syndecan-1 levels with the progression of osteoclastogenesis, suggesting syndecan-1 as a novel modulator of the RANKL and OPG balance in RA.

Independently of CSF-1R, IL-34 signals *via* PTPRZ1 (65) (Figure 1). Human monocytes have been demonstrated to express PTPRZ1 (57). PTPRZ1 involvement has been linked to various inflammatory diseases such as inflammatory bowel disease (IBD) and lupus nephritis, where PTPRZ1 expression was found to correlate with IL-34, CSF1, and CSF-1R expression (9, 29, 66). The most recent study reported that IL-34-dependent PTPRZ1 activation ameliorates the effector phase of RA (9). In this study, PTPRZ signaling was shown to facilitate the removal of apoptotic neutrophils by macrophages preventing further escalation of synovial inflammation in RA conditions (9). However, the above-mentioned study (using the K/BxN-induced model) also reported a protective role of IL-34 in RA, contrasting with other studies (11, 43). The discrepancy may be due to the difference in how RA was induced (K/BxN-induced vs. collagen-induced murine RA model). Thus, further investigation on whether PTPRZ1 activation is protective throughout different models of RA is needed.

## IL-34 and Th17 cells

The positive correlation between the severity of RA and the number of synovial macrophages has been well-documented (67, 68) (Table 1). Certain alleles of class II human leukocyte antigen (HLA) antigen (e.g., DRB1-β chain) are associated with RA *via* increased disease occurrence and inflammatory activity (72, 73). This is strong evidence for the involvement of T helper (Th) cells in the pathogenesis of RA. Although the mechanisms by which Th cell activation occurs in the RA microenvironment are yet to be fully understood, a positive correlation between Th population levels and RA disease severity has been noted (74). Of the Th subsets, a subset that is predominantly associated with RA pathogenesis is the T helper 17 (Th17) cell.

**Table 1:** Effects of IL-34 on T lymphocyte activation.

**Affected Th response**	**Target cell**	**Effects of IL-34**	**References**
Th17	**Human**		
	PBMC	Increasing Th17 cell proliferation and IL-17 expression	(2)
	PBMC	Increasing IL-17 production	(19)
	Monocytes	Increasing Th17 cell proliferation through upregulating IL-6 expression by THP-1	(42)
	Macrophages	Expanding of F4/80^+^iNOS^+^ macrophage population; polarizing Th1 and Th17 cells	(43)
	FLS	Increasing Th17 cell proliferation through upregulating IL-6 expression by FLS	(4)
	FLS	Increasing IL-17 production	(44)
	**Mouse**		
	Macrophages	Increased numbers of Th1 and Th17 cells in *in-vitro* coculture of BM-derived macrophages with splenocytes	(11)
	N/A	Increased IL-17 production	(24)
Treg	**Human**		
	PBMC	No effects on Treg transcription factors or cytokine secretion	(2)
	PBMC	Suppression of effector T-cell proliferation; Expansion and potentiation of both CD4+ and CD8+ Tregs	(69)
	**Humanized mouse**		
	N/A	Mediating suppressive activity of Tregs in mixed lymphocyte reaction	(70)
	**Rat**		
	CSF1R+ cells (Macrophages?)	Mediating inhibition of alloreactive immune responses	(69)
	N/A	Mediating suppressive activity of Tregs *in vivo*	(71)

Previously, researchers have pinpointed Th17 cell differentiation, recruitment, and activation as a driver of the inflammatory response in RA pathogenesis (75). The impact of Th17 cells in RA pathogenesis is supported by the increased numbers of Th17 cells in RA patients as compared to healthy people (76). Earlier studies have shown a bias for Th17 cell polarization of naïve CD4^+^ T cells, which impose an inflammatory response in the surrounding environment (77). In addition, Th17 cells are being identified as osteoclastogenic subsets in autoimmune arthritis through cytokine secretion and RANKL expression (77).

Exploring the therapeutic potential of Th17 cell inhibition in RA has been studied previously, where IL-25 was found to suppress Th17 immune responses for attenuation of RA in a collagen-induced model (78). An *in vitro* study found that IL-34-induced macrophages possessed the capacity of converting certain memory CD4^+^ T cells into CCR4^+^CCR6^+^CD161^+^ Th17 cells, supporting the possibility that Th17 cells observed in RA disease conditions are activated through IL-34-induced macrophages (41). Congruently, a different study demonstrated that IL-34 participates in the differentiation of peripheral blood mononuclear cells (PBMC) to Th17 cells in RA patients (2). Stimulation of PBMC from RA patients with IL-34 showed an increased frequency of Th17 cells with upregulated expression of IL-17 (19). Moreover, such effects of IL-34 found in RA patients went unnoted from samples of healthy controls, supporting the notion that IL-34 effects on Th17 cell differentiation could be RA-specific (2). Human monocytes (THP-1) activated by IL-34 led to the secretion of IL-6 increasing Th17 cell numbers in RA (42). In line with this, IL-34 expression has shown involvement in Th1 and Th17 cell polarization through the expansion of certain macrophage populations in murine models (43). The increase of Th17 cell numbers has also been possible through IL-34-stimulated IL-6 secretion by RA FLS, such results are supported by the attenuation of Th17 cell production by IL-34-dependent RA FLS through the administration of IL-6 antagonists (4, 44). Similarly, myeloid-related protein, also highly abundant in RA SF, has been found to upregulate IL-6 production by FLS promoting Th17 differentiation (79). There is also evidence that IL-17 directly acts on FLS. Increased proliferation of RA FLS and inhibition of RA FLS apoptosis by IL-34 was neutralized when IL-17 inhibitor plumbagin was introduced (44). Th17 cells and IL-17 may participate in RA FLS survival by inhibiting the mitochondrial pathway of apoptosis (80). These collective results suggest a positive correlation between IL-34 and Th17 cell inflammatory response in RA.

A collagen-induced murine arthritis model study with additional IL-34 injection led to increased TNF-α and IL-17 mRNA expression in the synovial tissues, indicating a proinflammatory role for IL-34 *via* IL-17 production entailing aggravation of arthritis (24). This regulation of IL-17 production by IL-34 could also play a role in osteoclastogenesis (55). The same study reported that the enhanced expression of RANKL and OPG by FLS and PBMCs of RA patients by IL-34 was secondhandedly mediated by IL-17. This is in line with the known function of IL-17 as an osteoclastogenic factor for subsequent osteoporosis (81).

## IL-34 and Tregs

Currently, researchers have also been investigating the potential of IL-34 in the regulation of balancing different T-cell populations, with Tregs being proposed as another T-cell subset mediated by IL-34 stimulation (Table 1). Tregs are a subpopulation of T cells that maintain homeostasis and self-tolerance through the suppression of different immune responses, such as those induced by Th17 cells (82). Tregs are known to be critical for the prevention of autoimmunity, as observed in RA, possibly by inhibiting the cytokine production by other pro-inflammatory T-cell subsets, as such the immunomodulatory role of Tregs has been studied in different clinical conditions (70, 82, 83). Concerning the activity of RA Th17 cells, Treg-mediated suppression of Th17 activity was studied in an *in vitro* would healing model and a murine autoimmune neuroinflammation model (84, 85). Naïve and memory Tregs were able to suppress Th17 cells *ex vivo*, and also in the wound-healing model, Tregs suppressed the Th17 cell antifibrotic effects (84). In an experimental multiple sclerosis model, Tregs limited the access of Th17 cells to APCs and suppressed Th17 cell calcium signaling (85). While Treg involvement in the anti-inflammatory activity and resolution of RA can be inferred as higher numbers of Tregs and their inhibitory activity have been detected in clinical remission states of RA (86), reports have observed the immunoregulatory role of Tregs to be compromised in RA patients (86).

Recently, the imbalance between Th17 and Treg populations, which would potentiate an inflammatory response in the surrounding microenvironment, is being studied as one determinant of disease progression. A study reported an increased frequency of peripheral Th17 cells and elevated Th17 cytokines, such as IL-17, IL-23, IL-6, and TNF-α in RA patients compared to healthy individuals (87). This contrasted with the significant decrease in both the frequency of Tregs and respective Treg-related cytokine [e.g., transforming growth factor-beta (TGF-β)] levels (11, 87). Meyer et al. (11) found that the TGF-β expression in the synovium is reduced in IL-34 and syndecan-1 KO mice, indicating that the synovial TGF-β expression is IL-34-dependent (11). Likewise, an investigation of Th1, Th17, and Treg populations in the bone marrow of RA patients reported a significant increase in Th1 and Th17 cell frequencies, and, in parallel, a significant decrease in Tregs (88). Reestablishing the balance between Treg and Th17 cell populations in RA by inhibiting NLRP3 inflammasome has exhibited promising results, which alludes to a potential therapeutic role of IL-34 for rebalancing Treg and Th17 cell populations (89). The amelioration of the proinflammatory response in a murine collagen-induced arthritis model was conducted through IL-12 suppression for suppression of Th17 cells and increase of the Treg population, which provides support for Treg population expansion in the treatment of RA (90).

IL-34 has been proposed as a Treg-targeting cytokine in a study revolving around the transplant tolerance activity of IL-34-expanded Tregs (69). In line with this, a most recently published study reported that IL-34 deficiency impairs the suppressive function of CD4+ Tregs in rats and makes mice more prone to autoimmunity (71). Human macrophage exposure to IL-34 was verified to increase the numbers of CD8^+^ and CD4^+^ FOXP3^+^ Tregs. These specific Treg populations, as compared to Tregs not expanded *via* IL-34 addition, demonstrated improved suppressive action against anti-graft immune responses (69). Additionally, a study of graft-versus-host disease identified highly suppressive human Tregs producing IL-34 for their immunosuppressive activity (70). Notwithstanding, a study noted that IL-34 stimulation of PBMC isolated from RA patients did not affect differentiation or cytokine secretion of Tregs, despite IL-34 exposure of RA PBMCs increasing Th17 populations and IL-17 expression levels (2). Such results demonstrate the need for further investigation into the mechanisms by which IL-34 engages in the differentiation, proliferation, and activation of Tregs in RA.

## Discussion

RA is an inflammatory autoimmune disease with a multifaceted etiology. While certain aspects of RA etiology and pathogenesis are becoming better understood, there is still much to learn for the development of improved therapeutic approaches. When observing the inflammatory response of the RA synovium, a positive correlation between macrophages and RA disease severity is well-observed (7, 8). Additionally, different subsets of T cells are being attributed as RA biomarkers, whether due to their up- or down-regulation (87). Furthermore, these immune cells are shown to participate in RA-associated osteoclastogenesis (16, 91).

Increased levels of IL-34 are being detected in the serum and SF of RA patients (4, 17–23). As reports of IL-34 involvement in macrophage and T-cell polarization are being released, IL-34 presents itself as a promising therapeutic target for immune response and osteoclastogenesis regulation in RA. However, the mechanisms, by which IL-34 either aggravates or mitigates RA, are not clearly understood. Notably, two recent independent studies using IL-34 knockout mice showed contrasting effects of IL-34 on RA (9, 11). Such conflicting observations may have resulted from different pathological mechanisms given that one study used K/BxN serum injection (autoantibody transfer) (9) and the other collagen immunization (11) to induce RA in mice, recapitulating hypersensitivity reactions type II/III (antibody-mediated) and type IV (cell-mediated), respectively. As IL-34 is highly prominent in RA patients, there are growing expectations for its potential as biomarker and therapeutic target in RA. IL-34 is a promising biomarker (92). However, as RA etiology and pathogenesis are not uniform in humans, continuous investigations into the precise roles of IL-34-induced synovial macrophages in T-cell responses and careful and thorough assessment of IL-34 as a target in RA subsets are needed for future therapeutic strategies.

## Author contributions

All authors listed have made a substantial, direct, and intellectual contribution to the work and approved it for publication.

## Funding

This study was funded by the 2022 University Innovation Support Project at Handong Global University led by the Ministry of Education of the South Korea and Basic Science Research Program through the National Research Foundation of Korea (NRF) funded by the Ministry of Education of the South Korea (2021R111A3059820).

## Conflict of interest

The authors declare that the research was conducted in the absence of any commercial or financial relationships that could be construed as a potential conflict of interest.

## Publisher's note

All claims expressed in this article are solely those of the authors and do not necessarily represent those of their affiliated organizations, or those of the publisher, the editors and the reviewers. Any product that may be evaluated in this article, or claim that may be made by its manufacturer, is not guaranteed or endorsed by the publisher.

## Abbreviations

APC: antigen-presenting cell
CSF-1: colony-stimulating factor-1
FLS: fibroblast-like synoviocytes
IL-34: interleukin-34; Janus protein tyrosine kinase
OPG: osteoprotegerin
PBMC: peripheral blood mononuclear cells
PTPRZ1: protein tyrosine phosphatase receptor type ζ1
RA: rheumatoid arthritis
RANK: receptor activator of nuclear factor-κB
RANKL: RANK ligand
SF: synovial fluid
Th: T helper
Treg: regulatory T cell
TNF: tumor necrosis factor.
